# Statistical association of complete PYHIN gene family loss with flight and inverted roosting in bats

**DOI:** 10.3389/fimmu.2026.1791604

**Published:** 2026-05-28

**Authors:** Chanasei Ziemann, Madeline Streicher, Allyssa Sicks, Chi Zhang, Luwen Zhang

**Affiliations:** 1 School of Biological Sciences, University of Nebraska, Lincoln, NE, United States; 2 Nebraska Center for Virology, University of Nebraska, Lincoln, NE, United States

**Keywords:** AIM2, bats, flight, IFI16, innate immunity, inverted roosting, PYHIN

## Abstract

The PYHIN (pyrin and HIN domain–containing) gene family encodes central cytosolic DNA sensors, including AIM2 and IFI16, that activate inflammasome and type I interferon pathways during infection. While these pathways are critical for antiviral and antimicrobial defense, how ecological pressures shape their evolution remain unclear. The PYHIN gene family varies across mammals, and bats have completely lost all PYHIN genes. Here, we integrate phylogenomic, comparative genomic, and phylogenetically controlled analyses across more than 150 mammalian species to investigate PYHIN evolutionary dynamics. We show that PYHIN genes form a tightly linked genomic cassette within a conserved chromosomal interval flanked by SPTA1 and CADM3, a pattern we describe as an Anchored Gene Cluster Pulsation mechanism characterized by coordinated expansion, contraction, and loss. Across mammals, the genomic distance between these anchor genes correlates with PYHIN copy number. In bats, phylogenetic logistic regression identifies powered flight and inverted roosting as traits statistically associated with PYHIN loss, whereas echolocation and hibernation show no association. Olfactory receptor genes within the same region are retained, indicating targeted loss of DNA-sensing immune genes rather than generalized genome contraction. These findings support a model in which bat immunity is associated with ecological specialization favoring immune tolerance and provide a framework for understanding immune gene family evolution as integrated genomic modules.

## Introduction

The PYHIN (pyrin and HIN domain-containing) gene family regulates innate immunity and inflammation by sensing cytosolic DNA and activating inflammasomes (1). Five members in humans, Absent in Melanoma 2 (AIM2), Interferon Gamma Inducible Protein 16 (IFI16), Myeloid Cell Nuclear Differentiation Antigen (MNDA), Pyrin and HIN Domain Family Member 1 (PYHIN1), and Pyrin Only Protein 3(POP3), coordinate responses to infection and stress via conserved PYD and HIN domains (1–3). Other animals, especially rodents, have additional PYHIN members, such as Interferon-inducible protein p203 (IFI203) (4, 5). AIM2 and IFI16 detect pathogenic or mislocalized DNA, triggering inflammasome or interferon responses, while POP3 modulates these pathways through PYD interactions (3). Despite their immune centrality, PYHIN genes show enigmatic evolutionary patterns. Bats uniquely lost PYHIN genes among mammals, while birds never acquired this mammalian innovation (6).

Despite their well-established immunological roles, PYHIN genes show striking and poorly understood variation across vertebrates (6, 7). Among mammals, the complete absence of PYHIN genes in bats is particularly notable given the critical role of these genes in inflammatory and antiviral defense in other species. This observation raises a fundamental immunological question: how can bats tolerate the loss of a major DNA-sensing pathway, and what evolutionary forces drove this extreme form of immune remodeling?

Understanding PYHIN evolution is especially relevant in the context of bat immunity. Bats can tolerate infections by certain viruses that are highly pathogenic in humans, a phenomenon that has led to their immune systems being described as exhibiting “super-immunity.” Powered flight has long been proposed as a key driver of bat immune specialization, as it imposes exceptional metabolic demands and increases the risk of inflammation-associated tissue damage. Other bat-associated ecological traits, such as inverted roosting and echolocation, have received far less attention. Importantly, the evolutionary relationships between these ecological traits and specific immune gene losses have not been systematically tested.

Here, we address these questions through comprehensive phylogenomic and comparative genomic analyses spanning more than 150 species of mammals, birds, reptiles, and fishes. We show that the PYHIN gene family emerged in therian mammals approximately 160–180 million years ago and subsequently underwent lineage-specific expansion, contraction, and complete loss. Importantly, the absence of PYHIN genes in monotremes reflects a different evolutionary scenario than that observed in bats. Because the PYHIN gene family originated after the divergence of monotremes, these species never acquired PYHIN genes. In contrast, bats inherited PYHIN genes from a therian ancestor and subsequently lost the entire gene family. This distinction highlights that absence of PYHIN genes across lineages can arise through fundamentally different evolutionary processes—failure to acquire versus secondary loss. We propose the Anchored Gene Cluster Pulsation Hypothesis, in which PYHIN genes evolve as a coordinated genomic cassette embedded within a conserved chromosomal framework, undergoing episodic gain and loss rather than gradual, gene-by-gene turnover. Importantly, phylogenetically controlled analyses identify powered flight and inverted roosting as the ecological traits most strongly associated with complete PYHIN loss in bats. Together, our findings provide an immunologically grounded framework for understanding how ecological pressures can drive large-scale remodeling of innate immune sensing pathways, resulting in immune systems that are uniquely adapted—though not universally enhanced—to specific physiological and environmental demands.

## Results

### Evolutionary origin and distribution of the PYHIN family

**Figure 1:**
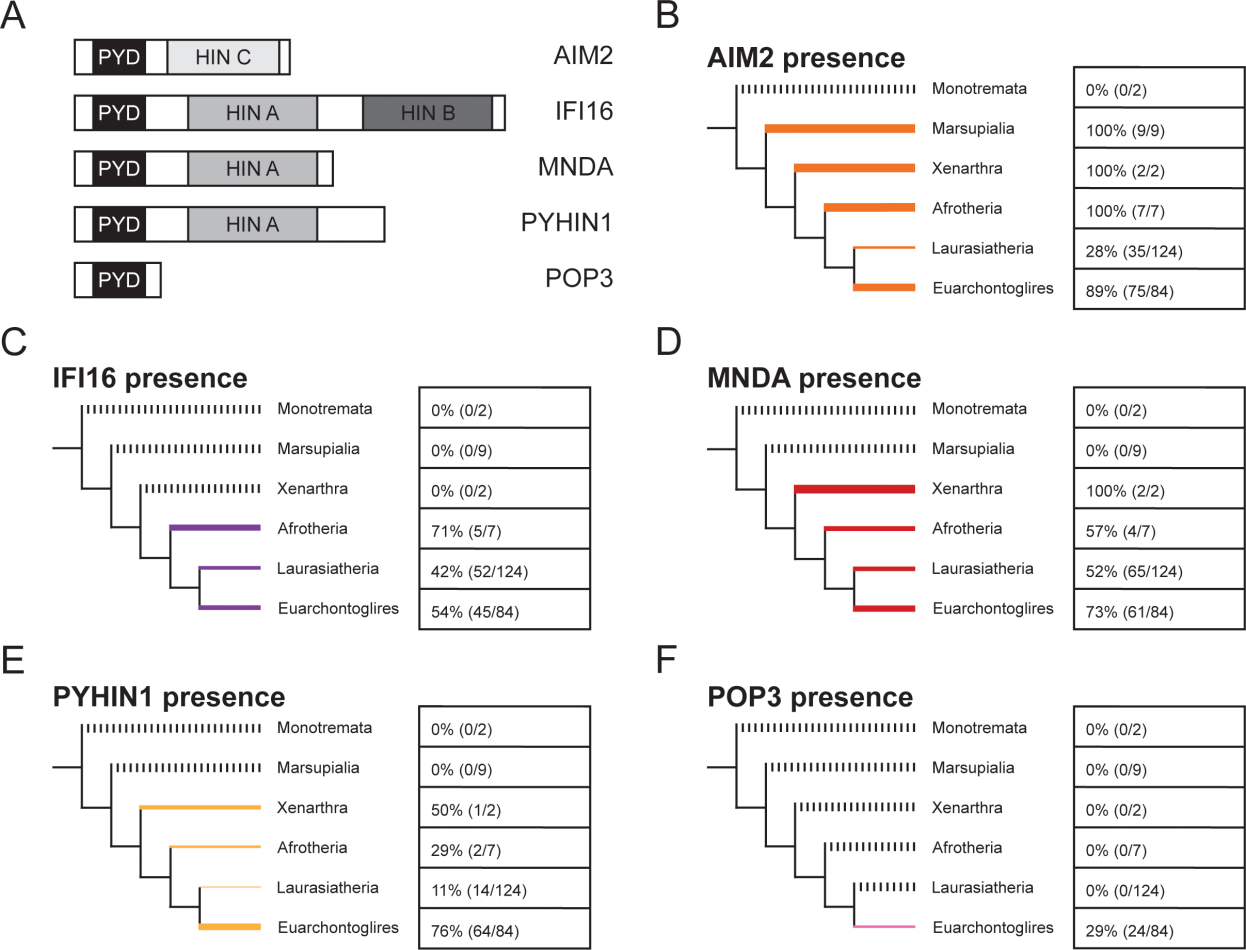
Evolutionary distribution of PYHIN family genes across major mammalian clades. **(A)** Schematic representation of domain structures for the five PYHIN family members in humans. Each protein contains a PYD domain and one or more HIN domains, except for POP3, which only has a PYD domain. **(B–F)** Phylogenetic distribution of individual PYHIN family members (AIM2, IFI16, MNDA, PYHIN1, and POP3) across major mammalian lineages. Simplified mammalian tree shows the four major superordinal clades. Sequence information is presented in **Supplementary Table 1**. Tree topology follows previous publications (8–11). The dashed lines represent not present in the described clades. Line thickness represents the percentage of species within each clade that contain the respective gene. Numbers in parentheses indicate the number of species with the gene relative to the total number of species examined in each clade, along with the exact percentage. Color coding is consistent across all panels: AIM2 (orange), IFI16 (purple), MNDA (red), PYHIN1 (yellow), and POP3 (pink).

We systematically searched for PYHIN protein coding sequences, identifying members through sequence similarity and phylogenetic analysis (see **Supplementary Table 1**). The distribution of PYHIN genes varies across mammalian lineages, with distinct presence/absence patterns characterizing different clades. Notably, as shown in **Figures 1A–F**, none of the five PYHIN family members (AIM2, IFI16, MNDA, PYHIN1, and POP3) were detected in monotremes (0% presence). In addition, AIM2 exhibits the broadest distribution across placental mammals, with 100% presence in Xenarthra (2/2), Afrotheria (7/7), and Marsupialia (9/9) and a high representation in Euarchontoglires (89%; 75/84). In contrast, POP3 has the most restricted distribution, being completely absent in most mammalian lineages except for Euarchontoglires (29%; 24/84). Our analysis revealed that the PYHIN gene family is a placental mammal-specific innovation; its distribution varies significantly across major mammalian clades.

### Hominoids retain a five-gene PYHIN repertoire

**Figure 2:**
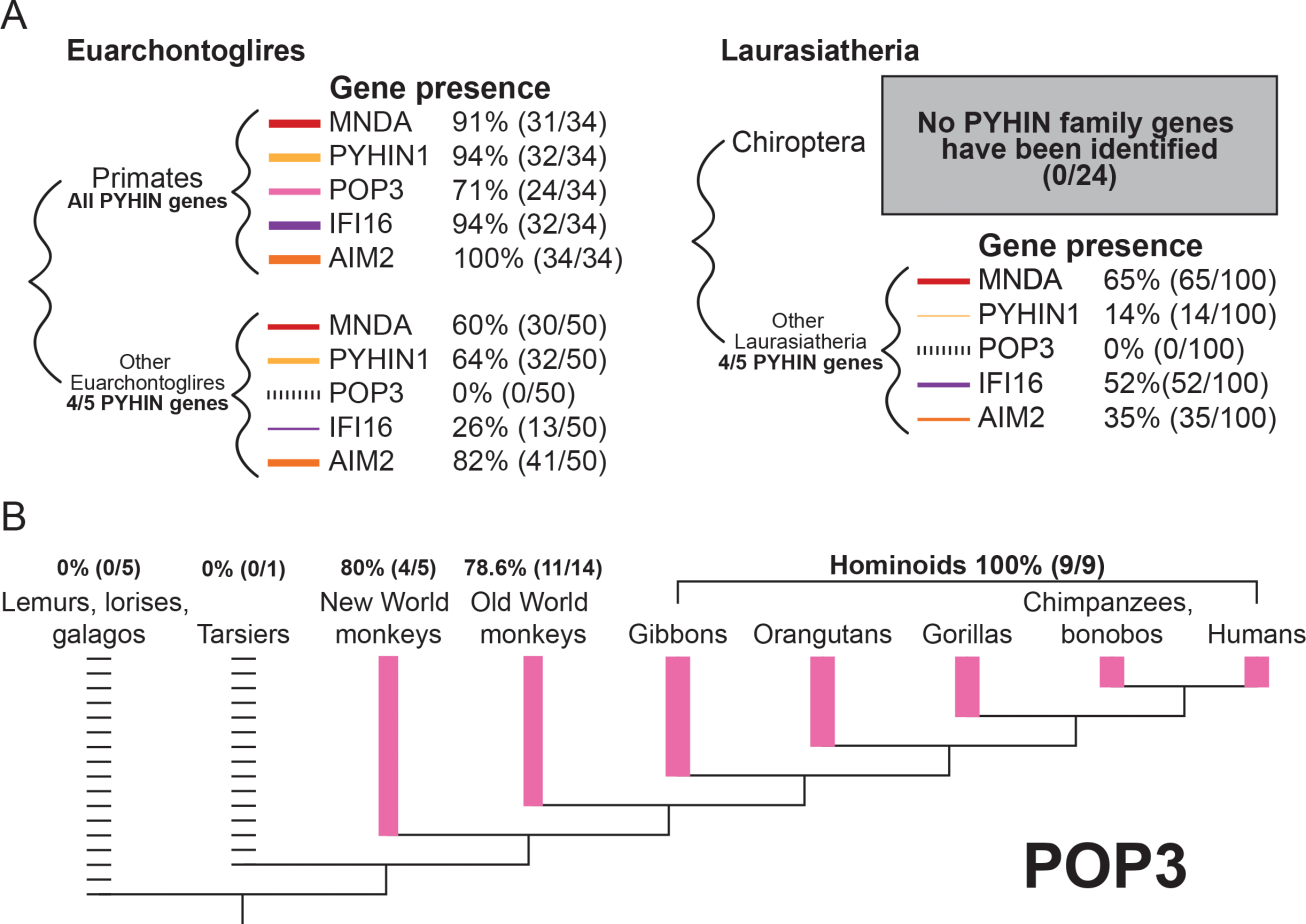
PYHIN family genes distribution in primates. **(A)** Left panel: PYHIN gene distribution in Euarchontoglires, highlighting complete retention of all five PYHIN genes in primates compared to partial retention in other Euarchontoglires. Right panel: PYHIN family gene distribution in Laurasiatheria, showing complete absence in Chiroptera (bats) and variable presence in other Laurasiatherian species. Color coding represents different PYHIN family members: MNDA (red), PYHIN1 (yellow), POP3 (pink), IFI16 (purple), and AIM2 (orange). The dashed lines represent not present in the described groups. Numbers in parentheses indicate the fraction of species containing each gene. **(B)** Distribution of POP3 gene across primate lineages, demonstrating their absence in galagoes (0%, 0/5) and tarsiers (0%, 0/1) (dashed lines), high prevalence in New-World monkeys (80%, 4/5) and Old-World monkeys (78.6%, 11/14), and universal presence in all hominoids (100%, 9/9). The taxonomic grouping of primates follows (12).

Our analysis of PYHIN gene distribution across mammals revealed the differences among taxonomic groups (**Figure 2A**). Primates showed conservation of all five PYHIN genes: AIM2 (100%), IFI16 (94%), MNDA (91%), PYHIN1 (94%), and POP3 (71%), distinguishing them from other Euarchontoglires, which exhibited greater variability and a complete absence of POP3. In contrast, bats (Chiroptera) lost all PYHIN genes, with none detected across 24 species as reported (6) (**Figure 2A**, right panel).

The POP3 protein contains the Pyrin domain but lacks the HIN domain, also called Pyrin Domain Containing 5 (PYDC5). It is sometimes considered a variant within the broader family context. POP3 distribution followed a phylogenetic gradient (**Figure 2B**). It was absent in prosimians (0%, 0/5) and tarsiers (0%, 0/1) but appeared frequently in anthropoids: New World monkeys (80%, 4/5) and Old World monkeys (78.6%, 11/14). Hominoids retained POP3 universally (100%, 9/9). Furthermore, phylogenetic analysis reveals it is indeed a hominoid-specific factor (**Supplementary Figure 1**). In addition, all hominoids have four other genes in the PYHIN family (**Supplementary Table 1**).

**Figure 3:**
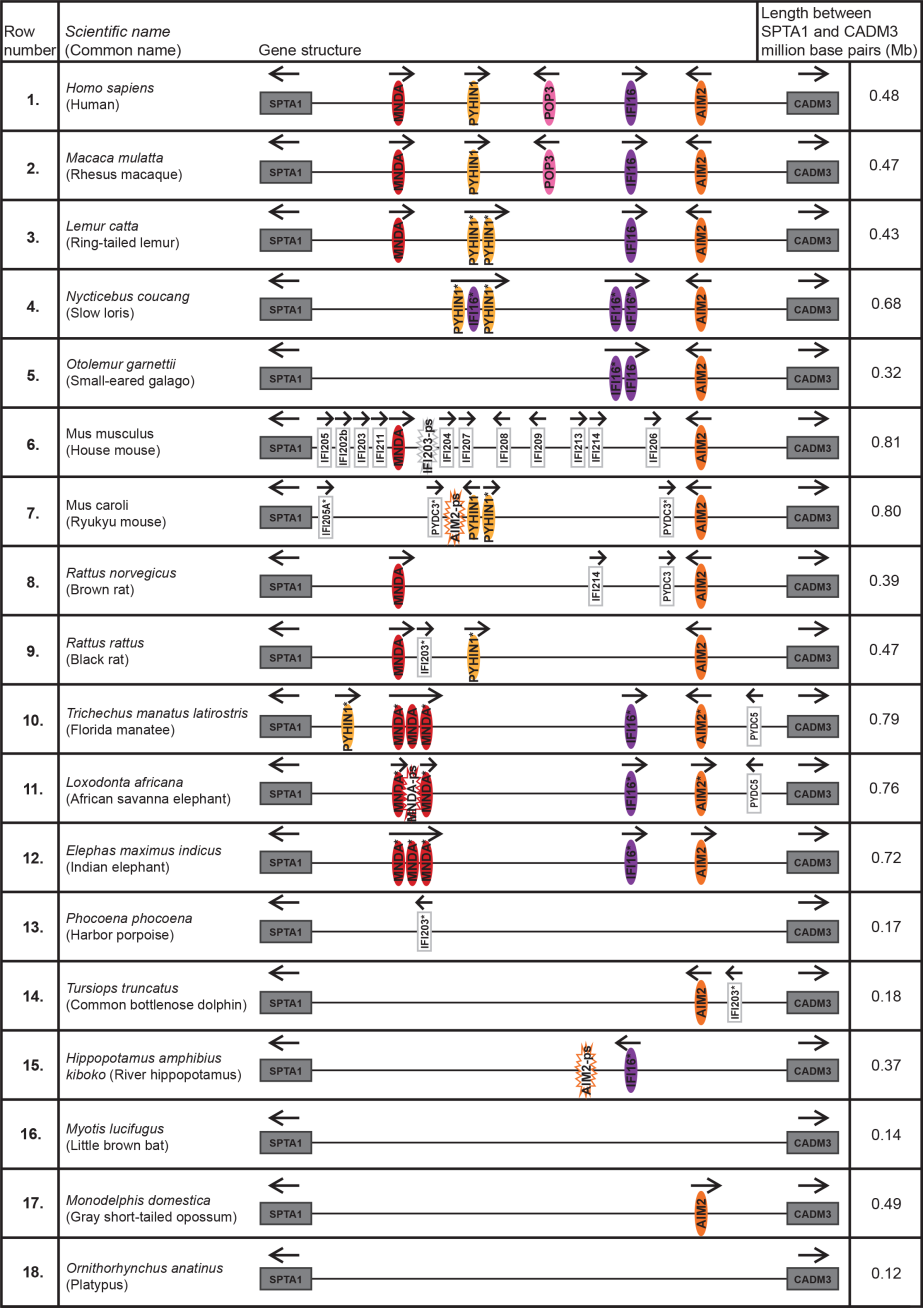
Syntenic analysis of SPTA1 and CADM3 genomic regions across vertebrate species. The genomic organization and orientation of SPTA1, CADM3, and the intervening genes are shown for representative vertebrate species. Arrows indicate the transcriptional direction of genes. Gray boxes represent SPTA1 and CADM3, while colored ovals denote different gene families located between them, with similar colors indicating gene family relationships. Rodent-specific paralogs are shown as open rectangles. Pseudogenes are labeled with *ps* after the gene name. Other lineage-specific genes are also indicated. Note that gene spacing is not drawn to scale. The right column represents the distance between SPTA1 and CADM3 in million base pairs (Mb).

Our syntenic analysis further revealed distinct PYHIN gene arrangements across primates (**Figure 3**, lanes 1–5). Humans and rhesus macaques (lanes 1–2) maintained conserved structures, while prosimians showed greater variation: ring-tailed lemurs (lane 3) retained the core but duplicated PYHIN1 and lacked POP3; slow lorises (lane 4) had multiple IFI16 duplications and two PYHIN1-like genes; and the small-eared galago (lane 5) exhibited the most divergence, lacking MNDA, PYHIN1, and POP3, but retaining duplicated IFI16-like genes and AIM2. These findings highlight the dynamic evolution of PYHIN genes in primates.

### The SPTA1-CADM3 region as an evolutionary hotspot for PYHIN dynamics

Spectrin alpha chain, erythrocytic 1 (SPTA1) maintains the biconcave shape of red blood cells, while Cell Adhesion Molecule 3 (CADM3) facilitates cell-cell adhesion and neural development. They are located on the same chromosome and all PYHIN family genes are located in the region (**Figure 3**).

Gene deletion is the most striking phenomenon in the region. Bats have lost all PYHIN genes while retaining the anchor genes (lane 16), whereas cetaceans (e.g., harbor porpoise, bottlenose dolphin) retain only one or two IFI203-like genes (lanes 13–14).

Gene duplication is most pronounced in mice, with *Mus musculus* expanding to at least 13 PYHIN members (**Figure 3**, lane 6), including lineage-specific paralogs like IFI202b, IFI204, and IFI207-IFI214—far exceeding the five found in humans (7, 13).

Pseudogenization also happens during PYHIN evolution. MNDA is a pseudogene in the African savanna elephant (lane 11), while AIM2 is pseudogenized in the hippopotamus (lane 15), and IFI203 in the mouse (lane 6).

Lineage-specific diversification further drives PYHIN evolution. MNDA-like expansions occur in Florida manatees and elephants (lanes 10-12), while PYHIN1 diversification is prominent in the ring-tailed lemur (lane 3). Novel IFI203-like genes in cetaceans exhibit modified domain structures, diverging from ancestral forms (lanes 13-14).

Even closely related species show marked variation. House and Ryukyu mice (lanes 6-7) and brown and black rats (lanes 8-9) have distinct PYHIN repertoire despite recent divergence. Similarly, African and Indian elephants (lanes 11-12) exhibit subtle but unique PYHIN arrangements.

These patterns of gene birth, expansion, contraction, and loss underscore the SPTA1-CADM3 region as an evolutionary hub for PYHIN innovation, with genomic plasticity constrained only by its conserved anchor genes.

### Establishment and conservation of the SPTA1-CADM3 genomic framework

To explore the genomic context of PYHIN gene evolution, we examined the evolutionary history of its flanking genes, SPTA1 and CADM3. SPTA1 has a homology to SPTAN1 (Spectrin Alpha, Non-Erythrocytic), while CADM3 belongs to the immunoglobulin superfamily.

**Figure 4:**
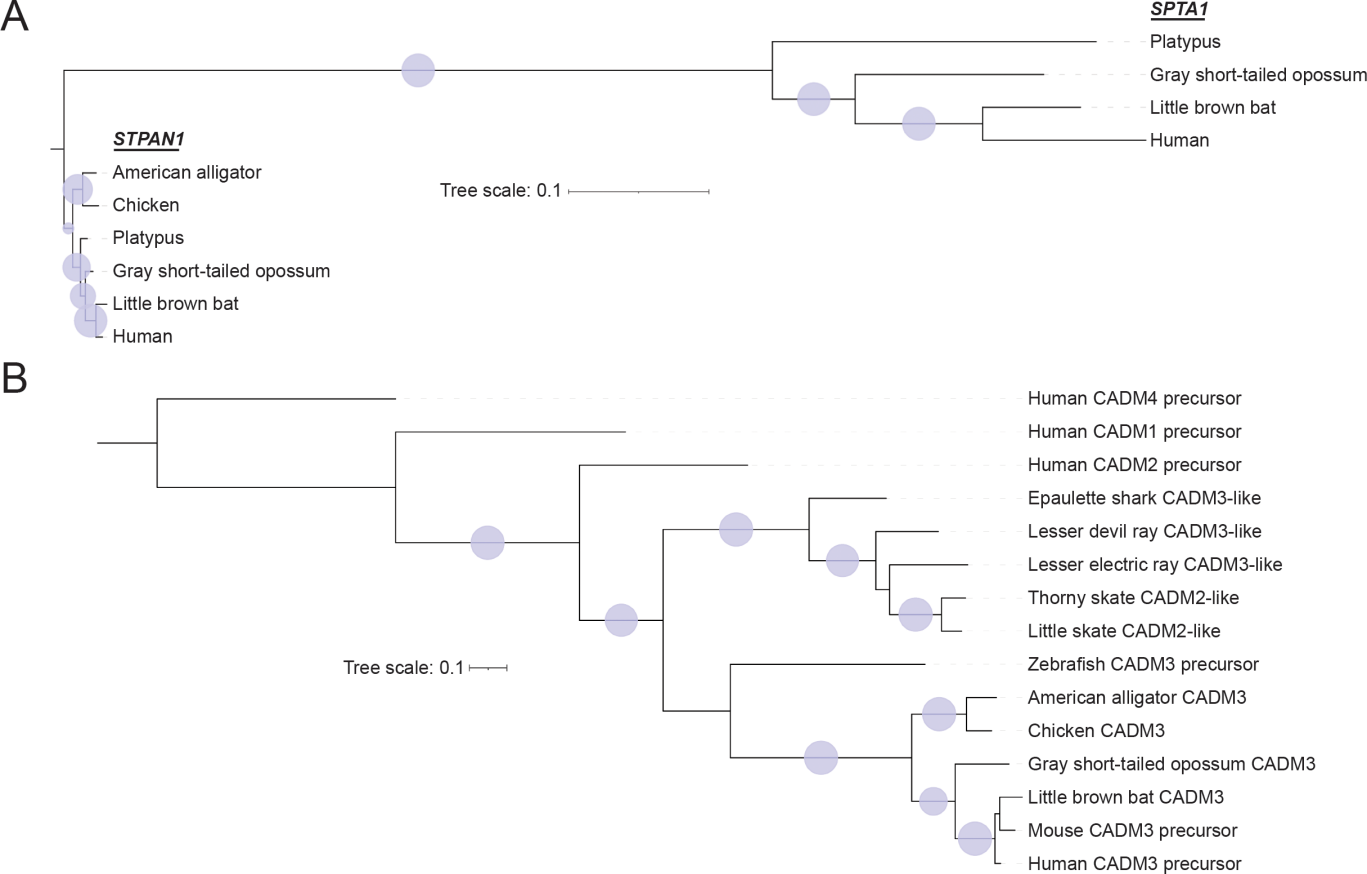
Evolutionary conservation of SPTA1 and CADM3 genes across vertebrates. **(A)** Phylogenetic relationships among SPTA1 and SPTAN1 protein families. The maximum likelihood phylogeny was reconstructed using protein sequences from representative vertebrate species. Internal nodes supported by 80% or higher in both ultrafast bootstrap and SH-aLRT branch tests are marked by gray dots, with dot size corresponding to ultrafast bootstrap values (80%−100%). The tree scale represents 0.1 substitutions per site. **(B)** Phylogenetic relationships among CADM3 protein families. Human CADM1–4 is labeled as hCADM1–4, while CADM3 orthologs in other species are labeled according to NCBI’s nomenclature. The same phylogenetic methods as in Panel A were used. The tree scale represents 0.1 substitutions per site, with node support indicated by bootstrap values (80–100%, represented by circle size). The sequences used to construct the tree are listed in **Supplementary Table 3**.

Our phylogenetic analysis (**Figure 4A**) shows that SPTA1 emerged in monotremes (e.g., *Ornithorhynchus anatinus*, platypus) and has persisted across all examined mammals. In contrast, CADM3 originated at the split between bony fish and cartilaginous fish (**Figure 4B**). The phylogenetic tree reveals a clear separation between the cartilaginous fish CADM protein coding sequences (e.g., epaulette shark and lesser devil ray) and the CADM3 orthologs found in fish and tetrapods (mammals, reptiles, and amphibians). We could not locate CADM3-like sequences in non-vertebrate lineages (data not shown). This topology, supported by high bootstrap values (>90%), indicates that CADM3 likely represents a vertebrate’s evolutionary innovation (**Figure 4B**).

While SPTA1 and CADM3 evolved along distinct paths, our data suggest they have remained on the same chromosome throughout evolution (**Figure 3**; **Supplementary Tables 1**, **2**). The presence of linked SPTA1-CADM3 genes in monotremes (which lack PYHIN genes) and the complete PYHIN deletion in bats (while retaining SPTA1-CADM3 linkage) demonstrates that this genomic framework was established prior to PYHIN emergence. A few exceptions exist (**Supplementary Table 2**; bottom section); however, these are based on preliminary sequence data and do not have verified chromosome assignments. Based on data in **Figures 3**, **4**, SPTA1 and CADM3 were established in proximity on the same chromosome before the divergence of Prototheria (monotremes) and Theria (marsupials and placental mammals).

### Genomic distance and PYHIN retention in the SPTA1-CADM3 region

**Figure 5:**
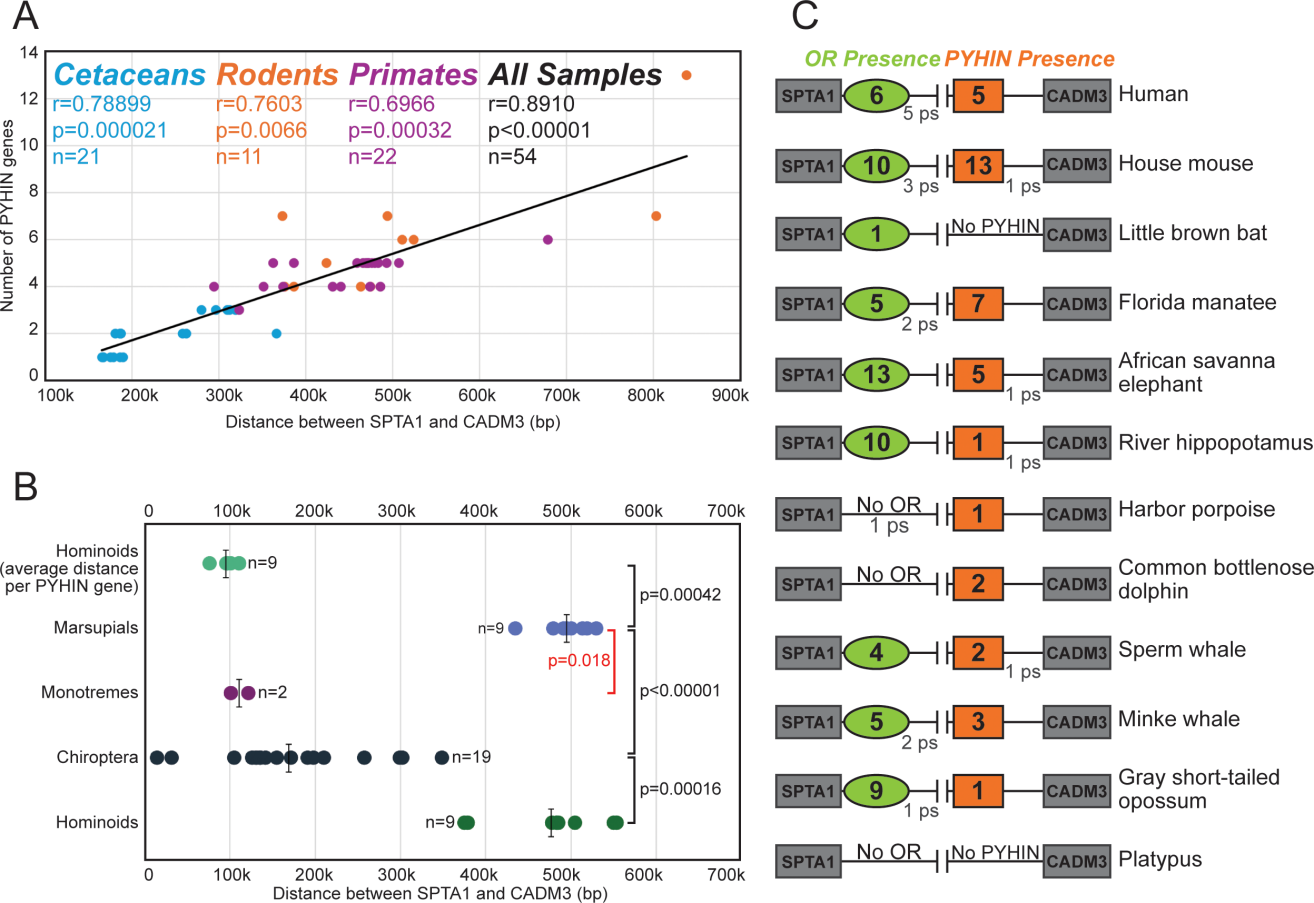
Genomic distance analysis of the SPTA1-CADM3 region and its relationship with PYHIN gene content. **(A)** Correlation analysis between SPTA1-CADM3 genomic distance and PYHIN gene count. A scatter plot shows a strong positive correlation in all the animal examined (black line) between the distance separating SPTA1 and CADM3 (x-axis, in base pairs) and the number of PYHIN genes (y-axis). The three groups: rodents (orange color), cetaceans (blue), and non-hominoid primates (purple) are also positively correlated (insert). Pearson correlation coefficient, number of data points, and statistical significance (*p*-values) are provided. Statistical significance was determined using an online calculator (see Methods for details). The sequences used to calculate the distances are listed in **Supplementary Table 3**. To account for phylogenetic dependence, phylogenetic generalized least squares (PGLS) analysis using the species tree yielded significant positive correlations for cetaceans (r = 0.7752, *p* = 0.0040), rodents (r = 0.7603, *p* = 0.0066), primates (r = 0.7462, *p* = 0.0002), and across all samples (r = 0.8931, *p* < 0.00001). **(B)** Comparative analysis of SPTA1-CADM3 distances across mammalian groups. The plot compares distances (in base pairs) between hominoids (average distance per PYHIN gene), marsupials (total distance with only one PYHIN gene), monotremes (total distance with no PYHIN genes), chiropterans/bats (total distance with no PYHIN genes), and hominoids (total distance containing five PYHIN genes). Differences between two groups were analyzed using the Mann-Whitney U Test for a two-tailed hypothesis. A permutation test (using the perm.test function in R) was conducted to compare monotremes and marsupials due to the limited monotreme data (n = 2). Because of the small sample size, conclusions about SPTA1-CADM3 distances between monotremes and marsupials (indicated by the red line) should be considered provisional. Statistical significance between groups is indicated by p-values. The sequences used to calculate the distances are listed in **Supplementary Table 3**. PGLS analysis yielded consistent patterns of group differences: hominoids (average distance per PYHIN gene) v.s. marsupials (p<0.0001), marsupials vs monotremes (p<0.0001), marsupials vs chiropterans (p= 0.0012), and chiropterans vs hominoids (p= 0.0012). **(C)** Comparative genomic organization of olfactory receptor (OR) and PYHIN genes within the SPTA1-CADM3 region across representative mammalian species. The schematic illustrates the conserved SPTA1 and CADM3 genes (gray boxes on the left and right, respectively) as anchor genes. The numbers within the colored ovals represent functional genes: OR genes (green) and PYHIN genes (orange/red). The number of pseudogenes is indicated by a number followed by “ps.” Note gene spacing is not drawn to scale.

The distance between SPTA1 and CADM3 varies systematically across mammals and apparently correlates with PYHIN gene retention and deletion (**Figure 3**). To address this issue further, three groups of animals (rodents, cetaceans, and non-hominoid primates) were analyzed, and their genomes showed a strong correlation between SPTA1-CADM3 distance and PYHIN gene count (*r* = 0.89*, p* < 0.00001) (**Figure 5A**, black line). In addition, rodents, cetaceans, and non-hominoid primates also show positive correlation among themselves (**Figure 5A**, colored inserts). Hominoid data were omitted from the correlation analysis because they exhibit no variation in PYHIN gene counts, which would not contribute to assessing a correlative relationship with the SPTA1-CADM3 genomic distance. Monotremes, which lack PYHIN genes, have shorter SPTA1-CADM3 distances (~110kb vs. ~495kb in marsupials, *p* = 0.018). However, since sequence data are available for only two monotreme species, this conclusion should be interpreted with caution. Despite having distances similar to those of hominoids, marsupials retain only AIM2 (**Figure 5B**). Also, bats exhibit even shorter distances, reflecting complete PYHIN loss (**Figure 5B**). Overall, SPTA1-CADM3 distance correlates strongly with PYHIN content across mammals.

SPTA1-CADM3 genomic region in mammals exhibits parallel evolution of olfactory receptor (OR) gene families, reflecting species-specific ecological adaptations. (**Figure 5C**). These patterns underscore the evolutionary interplay between sensory and immune functions shaped by ecological pressures.

#### SPTA1-CADM3 is a minimal genomic boundary for co-evolving sensory and immune genes

CADM3 is frequently found adjacent to AIM2 across various species, with examples such as the southern two-toed sloth (*Choloepus didactylus*) showing overlapping transcripts (Gene ID: 119526496; updated 19-Nov-2025). Similarly, SPTA1 is intimately associated with OR genes. In pigs (*Sus scrofa*), compelling evidence of this integration is seen through overlapping transcripts between PYHIN and OR genes (Gene ID: 100154852, updated 1-Mar-2025), and also between some OR and SPTA1 transcripts (Gene ID: 100152068, updated 1-Mar-2025). All those observations support that the SPTA1-CADM3 genomic region serves as a crucial and minimal boundary encompassing the dynamic co-evolution of both PYHIN and OR genes.

#### Flight and inverted roosting strongly associated with lineage-specific loss of PYHIN genes

Flight has long been proposed as a major determinant of bat immune biology; however, this idea has not been subjected to rigorous statistical testing, particularly in a phylogenetic context that accounts for shared evolutionary history. In addition, inverted roosting, a near-universal behavior among bats, has received little attention as a potential immunological selective pressure.

To formally evaluate the contributions of these traits, we implemented a phylogenetically controlled comparative framework that jointly analyzes flight, inverted roosting, and other bat-associated ecological traits across mammals (**Supplementary Table 4**). By explicitly accounting for phylogenetic non-independence among species, we test whether these traits are statistically associated with variation in immune gene repertoire size.

**Figure 6:**
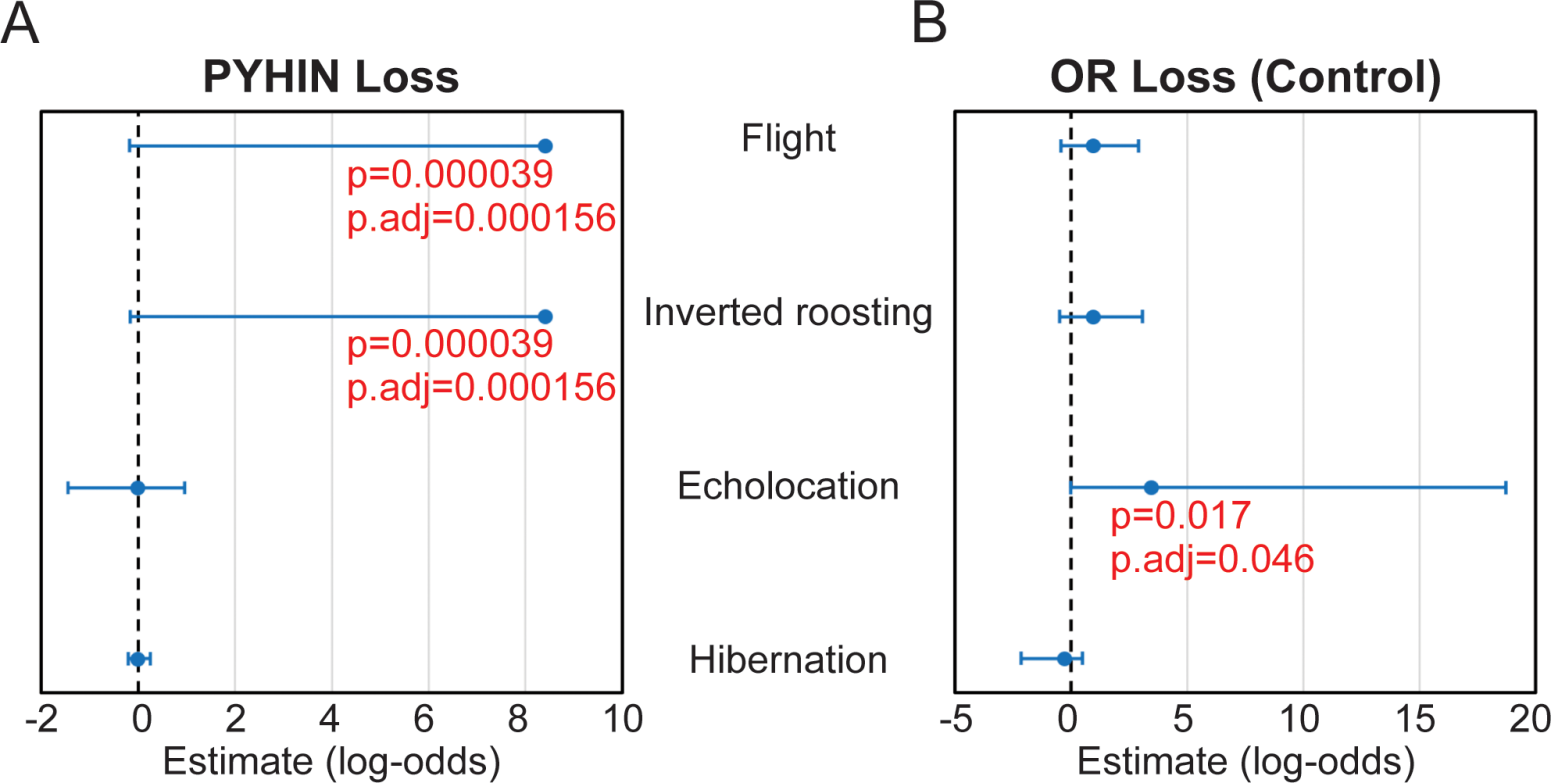
Flight and inverted roosting are strongly associated with PYHIN loss, with no parallel effect on OR gene loss. **(A)** Phylogenetic logistic regression estimates (± bootstrap 95% confidence intervals) showing the effects of four ecological traits—flight, inverted roosting, echolocation, and hibernation—on the probability of PYHIN gene loss across mammals. Blue circles represent model-estimated coefficients (log-odds of gene loss), and horizontal bars indicate uncertainty based on parametric bootstrapping. Flight and inverted roosting exhibit strong positive effects on PYHIN loss. Echolocation and hibernation show no detectable association, with estimates centered near zero. **(B)** Identical regression model applied to OR gene loss in the same region and used as an ecological and functional control. In contrast to PYHIN genes, neither flight nor inverted roosting is associated with OR loss, while echolocation shows a moderate association, reflecting known sensory trade-offs in echolocating mammals. **Supplementary Table 5** provides the full statistical results for all models, including regression coefficients, standard errors, bootstrap confidence intervals, significance values, and model diagnostics.

We identify a robust and highly specific association between the evolution of flight and inverted roosting behavior and complete loss of the PYHIN immune gene family (**Figure 6A**). Both traits show strong positive associations with PYHIN deletion (slope = 8.4, adjusted p = 0.0002). In contrast, other ecological traits commonly associated with bats, including echolocation and hibernation, show no detectable relationship with PYHIN dynamics (echolocation: slope = 0.000334, adjusted p = 0.9998; hibernation: slope = 0.000040, adjusted p = 0.9998). Parallel analyses of OR gene loss within the same genomic region likewise show no association with flight or inverted roosting (both slope = 0.951, adjusted p = 0.627; **Figure 6B**). Echolocation, however, exhibits a statistically significant association with OR reduction (slope = 3.454, adjusted p = 0.0460), consistent with established sensory trade-offs in echolocating mammals (**Supplementary Table 5**). Together, our phylogenetic logistic regression identifies a strong statistical association between the evolution of flight and inverted roosting and complete loss of the PYHIN gene family; however, because these traits are largely restricted to the Chiroptera lineage, the individual contributions of trait-specific versus lineage-specific effects cannot be fully separated (**Supplementary Tables 4**, **5**).

## Discussion

### Flight, inverted roosting, and DNA-sensing immune gene loss

Fossil evidence indicates that both flight and inverted roosting arose early in chiropteran evolution and have been maintained as an integrated biomechanical system (14–17). Our phylogenetically controlled analyses identify flight and inverted roosting as the strongest statistical association factors of complete PYHIN loss across mammals (**Figure 6**). Other traits commonly invoked in discussions of bat immunity—such as echolocation or hibernation—show no association with PYHIN dynamics. Because all extant bats fly and almost all roost inverted, and because genomic data are lacking for the few exceptional bat species, our analyses cannot statistically disentangle the individual contributions of these two traits. Nonetheless, their tight evolutionary and functional coupling is consistent with the hypothesis that the ecological transition to powered flight, together with its associated inverted roosting, may have influenced the evolution of DNA-sensing immune genes.

From an immunological standpoint, chronic or inappropriate activation of PYHIN-dependent pathways under conditions of frequent DNA damage would be maladaptive. Persistent inflammasome activation can drive tissue injury, systemic inflammation, and reduced fitness. Complete deletion of the PYHIN gene family eliminates not only functional signaling but also the risk of leaky or dysregulated activation that can arise from partially functional or pseudogenized genes. Thus, PYHIN loss in bats may represent a genomic pattern consistent with reduced inflammatory liability under flight and inverted roosting-associated physiological constraints.

### Loss of DNA-sensing immune genes in bats suggesting potential immune tolerance

Bats are divided into two major suborders, Yinpterochiroptera and Yangochiroptera, which diverged early in chiropteran evolution. The absence of PYHIN genes in both lineages indicates that this loss occurred in their common ancestor prior to this split, supporting the view that bat immune system evolution reflects a shared ecological adaptation rather than enhanced antiviral specialization. The loss of PYHIN genes provides a mechanistic explanation for several hallmark features of bat immunity. Bats are well known for their ability to tolerate viral infections that are highly pathogenic in other mammals, including humans (18–21). Reduced DNA-triggered inflammasome signaling may contribute to the observed viral tolerance by dampening excessive inflammation and immunopathology. Importantly, this does not imply superior or broadly enhanced immunity. Rather, bats appear to have evolved a genomic immune profile consistent with minimizing inflammatory self-damage in the context of extreme metabolic and biomechanical demands.

This specialization entails trade-offs. While reduced inflammasome activity may favor viral tolerance, it may also compromise defenses against certain pathogens. The high susceptibility of bats to fungal pathogens such as *Pseudogymnoascus destructans*, the causative agent of white-nose syndrome, is consistent with this view (22, 23). Thus, bat immunity is neither “super” nor deficient; it is uniquely tuned to the ecological realities of flight and inverted roosting.

### Anchored pulsatile evolution as a framework for immune gene remodeling

**Figure 7:**
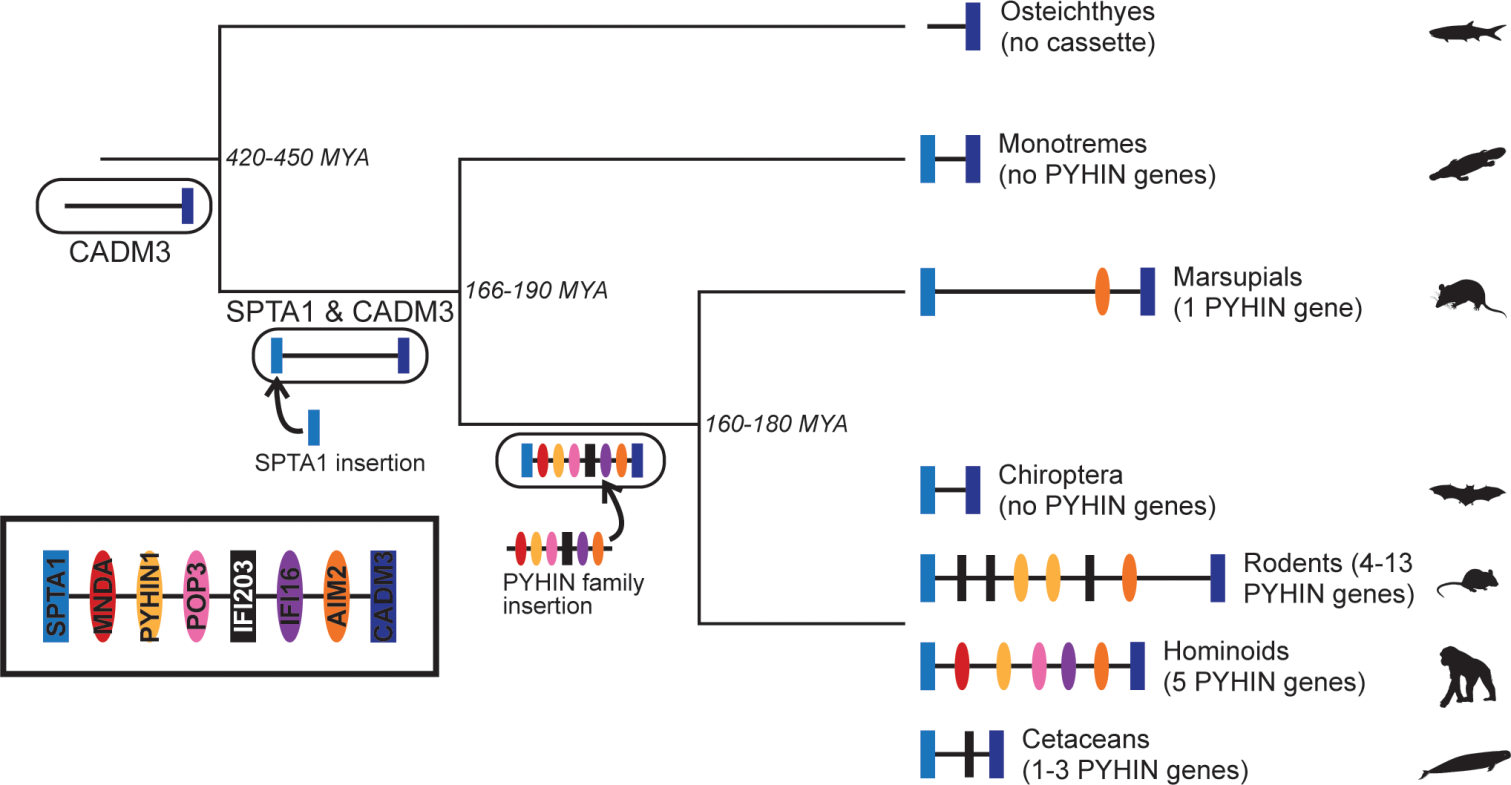
Pulsatile PYHIN evolution via the anchored gene cluster pulsation hypothesis. The PYHIN gene family evolves as a genomic cassette embedded within a stable chromosomal framework flanked by the anchor genes SPTA1 and CADM3. According to the Anchored Gene Cluster Pulsation Hypothesis, immune gene content within this conserved interval undergoes episodic, cassette-level expansion, contraction, and deletion (“pulsation”) rather than continuous gene-by-gene turnover. Evolutionarily, the common ancestor of bony fish and tetrapods retained only CADM3 (420–450 MYA). The SPTA1–CADM3 anchor framework was subsequently established in early mammals (166–190 MYA), followed by the emergence of the PYHIN gene family in therians (160–180 MYA). After this origin, lineage-specific pulsation patterns are observed: marsupials retain a single PYHIN gene; bats exhibit complete PYHIN deletion accompanied by interval contraction; and rodents, hominoids, and cetaceans display variable PYHIN expansion and retention. Across multiple mammalian lineages, the genomic distance between SPTA1 and CADM3 correlates strongly with PYHIN gene copy number, consistent with coordinated cassette-level remodeling. Colored bars represent individual PYHIN family members and related genes. Relative distances between anchor genes are shown for comparison, but gene spacing is not drawn to scale. Animal icons highlight lineage-specific patterns, and species are grouped into broad ecological niches based on published natural history accounts (24–26).

Having established the immunological and ecological context, our data support a broader evolutionary framework to explain how such radical immune remodeling can occur. The PYHIN genes are embedded within a conserved genomic interval flanked by SPTA1 and CADM3, which remains intact across mammals regardless of PYHIN content. Within this stable framework, the PYHIN family undergoes coordinated expansion, contraction, and deletion—a pattern we describe as the Anchored Gene Cluster Pulsation Hypothesis (**Figure 7**).

Multiple independent lines of evidence support this hypothesis. First, unlike classical birth–death models, which assume continuous, gene-by-gene duplication and loss across the genome, PYHIN evolution is constrained within a stable SPTA1–CADM3 genomic framework that persists across mammals. This interval shows stepwise assembly—CADM3 arising early in vertebrates (420–450 MYA), SPTA1 emerging later in mammals (166–190 MYA), and the full PYHIN cassette appearing in therians (160–180 MYA)—followed by coordinated expansion and contraction (**Figure 7**). Second, across multiple mammalian lineages, including rodents, cetaceans, and non-hominoid primates, the physical distance between SPTA1 and CADM3 strongly correlates with PYHIN gene copy number, consistent with cassette-level gain and loss rather than scattered gene turnover (**Figure 5**). Third, lineage comparisons further support discrete pulses of evolution. Monotremes exhibit a compact interval lacking PYHIN genes; however, this absence reflects their divergence prior to the origin of the PYHIN gene family and therefore represents a failure to acquire, rather than gene loss. In contrast, bats inherited PYHIN genes from a therian ancestor and subsequently lost the entire gene family, representing a true case of complete secondary loss. This distinction is critical for interpreting evolutionary patterns across mammals, as similar absence states can arise through fundamentally different processes—ancestral absence versus lineage-specific deletion (**Figure 7**). Fourth and finally, modularity within the region—exemplified by independent retention or loss of PYHIN and olfactory receptor genes—demonstrates that this anchored architecture permits selective, pulse-like remodeling of functionally related gene families without destabilizing neighboring loci (**Figures 3**, **5**).

Although PYHIN genes show dynamic gain and loss across mammals, the observed patterns are not fully explained by a simple birth–death model. The strict confinement of PYHIN genes within the SPTA1–CADM3 interval, the strong correlation between interval size and gene number, and the coordinated, complete deletion in bats collectively indicate cassette-level remodeling. We therefore propose that the Anchored Gene Cluster Pulsation model provides the most parsimonious explanation for these patterns, while incorporating local stochastic processes within a constrained genomic framework.

Importantly, this framework explains how bats could completely delete a major immune-sensing pathway while retaining neighboring gene families, including olfactory receptors. The specificity of PYHIN loss argues against generalized genome contraction and instead highlights targeted immune remodeling driven by selection against inflammatory cost rather than loss of immune competence.

### Implications for comparative and evolutionary immunology

Together, our findings place bat immunity within a broader immunological principle: immune systems evolve under competing pressures of protection and self-damage. In bats, the balance has shifted decisively toward tolerance, facilitated by the complete removal of DNA-sensing inflammasome pathways. The Anchored Gene Cluster Pulsation Hypothesis provides a mechanistic explanation for how such shifts can occur rapidly and coordinately at the genomic level, in which genes undergo episodic expansion and contraction within fixed genomic boundaries. This organization allows immune gene content to change rapidly in response to ecological pressures while preserving overall genomic structure (**Figures 3**, **7**). Our research underscores the importance of ecological context in interpreting immune gene repertoires. Apparent immune “losses” may represent adaptive refinements rather than deficiencies. More broadly, our study highlights how genome architecture can enable large-scale immune innovation, allowing mammals to explore diverse ecological niches while maintaining overall genomic stability.

## Materials and methods

### Searching of PYHIN, CADM3, and SPTA1 Protein-Coding Sequences

The coding sequences of the human PYHINs, CADM3, and SPTA1 genes were used as the queries (see **Supplementary Table 1** for the accession numbers). BLASTP protein similarity search es were performed against the nonredundant protein database at the National Center for Biotechnology Information (NCBI) with default options. When several isoforms were available for a gene, one isoform whose protein-coding sequence was most like the human ortholog was selected. Protein sequences that were identical or partial (too short) were excluded. Subfamily classification of proteins is done based on the phylogenetic placement regardless of the annotation given in the NCBI database. In case of ambiguity, a reciprocal BLASTP similarity search was performed further.

### Phylogenetic analysis and classification of protein sequences

Multiple sequence alignment of the protein sequences was performed using MAFFT (ver. 7.526) with the E‐INS‐i iterative refinement method. The maximum likelihood (ML) phylogenetic analysis was performed using IQ‐TREE (ver. 1.6.12) with the options for automatic model selection and ultrafast bootstrap analysis and SH‐aLRT branch test for branch support analysis (both with 1000 replicates) (27, 28). The visualization of the phylogenies was performed using the Interactive Tree of Life website (ver. 6.9.1) (29).

### Comparative genomic analysis of the SPTA1-CADM3 genomic region

Genomic sequences containing SPTA1 and CADM3 genes were collected from various vertebrate species. Complete genome assemblies were accessed from NCBI public databases. SPTA1 and CADM3 genes were identified as conserved anchor points across all examined species. All genes located between these two anchors were systematically identified based on NCBI annotation.

PYHIN family genes (AIM2, IFI16, MNDA, PYHIN1, POP3) and their homologs were characterized through sequence similarity searches and phylogenetic analysis. Pseudogenes were identified and labeled (marked with “ps” in the figure). Genes were classified by family relationships (indicated by color coding in **Figure 3**). Orthologous relationships between genes were established using sequence similarity. Paralogs specific to certain lineages (particularly in rodents) were identified through comparative genomic analysis.

The orientation of each gene was determined using NCBI transcription information (shown by arrows in **Figure 3**). The relative positions of genes were mapped within the SPTA1-CADM3 region for each species. The genomic distance between SPTA1 and CADM3 was calculated for each species (shown in Mb in the right column of **Figure 3**). The OR genes are analyzed with the same procedure.

### Ecological classification

Mammalian lineages were classified into major ecological niches (aquatic, semi-aquatic, terrestrial/arboreal, aerial) based on habitat use, locomotor adaptations, and behavioral data compiled from published natural history references and primary literature sources. Aquatic species (e.g., cetaceans) are fully water-bound; semi-aquatic species (e.g., pinnipeds and monotremes) divide their time between aquatic and terrestrial environments; terrestrial/arboreal species inhabit land or trees; and aerial species (e.g., bats) are adapted for powered flight.

Ecological and behavioral trait data used in downstream analyses—including flight, echolocation, hibernation, and inverted roosting—were systematically compiled from the literature and are fully documented with source references in **Supplementary Table 4**. For each species, trait assignments were based on consensus descriptions from authoritative sources, including classic mammalian compendia (24–26) and additional primary studies where necessary.

Gene-specific data, including PYHIN gene presence/absence and annotation, were obtained from publicly available genome assemblies and curated databases (NCBI RefSeq and GenBank). All data sources for both ecological traits and genomic annotations are explicitly listed in **Supplementary Table 4** to ensure transparency and reproducibility.

Mammalian lineages were classified into major ecological niches (aquatic, semi-aquatic, terrestrial/arboreal, aerial) based on habitat use, locomotor adaptations, and behavioral data from published sources (24–26). Aquatic species (e.g., cetaceans) are fully water-bound; semi-aquatic species (e.g., some pinnipeds, monotremes) split time between land and water; terrestrial/arboreal species inhabit land or trees; and aerial species (e.g., bats) are adapted for flight. Social complexity was categorized using published data on group size, social structure, and interaction rates. Lineages with diverse ecological traits (e.g., rodents) were labeled as occupying “varied niches.”

### Statistical calculation

Pearson correlation coefficients were calculated with Microsoft Excel, and the statistical significance (p-values) of these coefficients was determined using online calculators for Pearson’s correlation, such as the one available at https://www.socscistatistics.com/pvalues/pearsondistribution.aspx. For analyzing differences between two groups, the Mann-Whitney U Test Calculator (https://www.socscistatistics.com/tests/mannwhitney/default2.aspx) was used for two tailed hypothesis. Additionally, permutation tests were conducted using the perm.test function in R. The standard threshold of p < 0.05 was considered statistically significant for all analyses. We used the Phylogenetic Generalized Least Squares (PGLS) framework in ape and nlme packages in R to include phylogenetic dependence in the correlation tests and Mann-Whitney U tests via the species tree obtained through the Open Tree of Life (30).

A reconciled gene and species tree was created using GeneRax (version 2.1.3, default parameters). Seventy-one species were selected for the gene tree and pruned from the mammalian species tree of Upham et al. (30) accessed through the Open Tree of Life.

Binary response variables of PYHIN gene loss or OR gene loss were tested against behavioral traits of flight, echolocation, hibernation, and head-down roosting. Trait and gene-loss datasets were analyzed using Firth’s phylogenetic logistic regression, using the phyloglm() function in the phylolm R package in R (version 2.6.5) under default parameters with 1000 bootstrap replicates. Each trait was tested in a separate model for each response variable. Adjusted p-values were computed using the Benjamini-Hochberg (BH) false discovery rate (FDR) method in the p.adjust() function. All sequence-based analyses in this study were conducted at the amino acid level unless otherwise specified.

## Data Availability

The original contributions presented in the study are included in the article/**Supplementary Material**. Further inquiries can be directed to the corresponding author.

## Supplementary material

Supplementary Figure 1 Phylogenetic relationships among POP3/PYDC5 protein families. POP3 nomenclature is used for primate PYDC5 proteins due to its high relation to the Human POP3 protein. The maximum likelihood phylogeny was reconstructed using protein sequences from representative vertebrate species. Internal nodes supported by 80% or higher in both ultrafast bootstrap and SH-aLRT branch tests are marked by gray dots, with dot size corresponding to ultrafast bootstrap values (80%−100%). The tree scale represents 0.1 substitutions per site. The sequences used to construct the tree are listed in **Supplemental Supplementary Table 2**.
