# Supplementary figures and images for: Statistical association of complete PYHIN gene family loss with flight and inverted roosting in bats
Source: Front Immunol. 2026 May 28;17:1791604. doi: 10.3389/fimmu.2026.1791604 (PMC13253634; doi:10.3389/fimmu.2026.1791604)

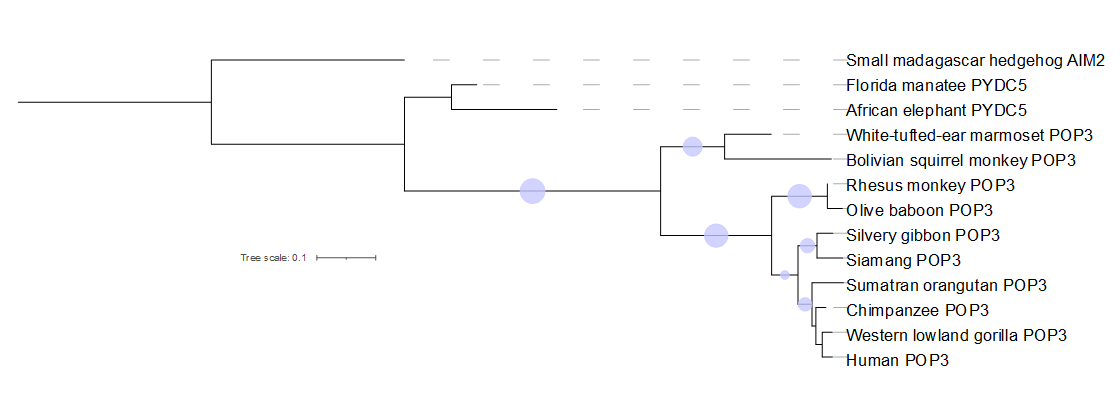

Supplement: Supplementary Figure 1 — Phylogenetic relationships among POP3/PYDC5 protein families. POP3 nomenclature is used for primate PYDC5 proteins due to its high relation to the Human POP3 protein. The maximum likelihood phylogeny was reconstructed using protein sequences from representative vertebrate species. Internal nodes supported by 80% or higher in both ultrafast bootstrap and SH-aLRT branch tests are marked by gray dots, with dot size corresponding to ultrafast bootstrap values (80%−100%). The tree scale represents 0.1 substitutions per site. The sequences used to construct the tree are listed in Supplemental Supplementary Table 2. [file Image1.tif]
